# Maresin-1 inhibits high glucose induced ferroptosis in ARPE-19 cells by activating the Nrf2/HO-1/GPX4 pathway

**DOI:** 10.1186/s12886-023-03115-9

**Published:** 2023-09-06

**Authors:** Yufei Li, Jieyu Liu, Xibo Ma, Xue Bai

**Affiliations:** 1 Ophthalmology Department, Zhongshan Hospital Affiliated to Xiamen University, No.201-209 Hubinnan Road, Siming District, 361004 Xiamen, China; 2 Endocrinology Department, Beijing Electric Power Hospital, 100073 Beijing, China; 3 Otorhinolaryngology Department, Jilin Province People’s Hospital, 130000 Changchun, China

**Keywords:** Maresin-1, Diabetic retinopathy, Ferroptosis, Nuclear factor erythroid 2-related factor 2

## Abstract

**Background:**

Maresin-1 plays an important role in diabetic illnesses and ferroptosis is associated with pathogenic processes of diabetic retinopathy (DR). The goal of this study is to explore the influence of maresin-1 on ferroptosis and its molecular mechanism in DR.

**Methods:**

ARPE-19 cells were exposed to high glucose (HG) condition for developing a cellular model of DR. The CCK-8 assay and flow cytometry were used to assess ARPE-19 cell proliferation and apoptosis, respectively. Furthermore, the GSH content, MDA content, ROS level, and Fe^2+^ level were measured by using a colorimetric GSH test kit, a Lipid Peroxidation MDA Assay Kit, a DCFH-DA assay and the phirozine technique, respectively. Immunofluorescence labelling was used to detect protein levels of ACSL4 and PTGS2. Messenger RNA and protein expression of HO-1, GPX4 and Nrf2 was evaluated through western blotting and quantitative real time-polymerase chain reaction (qRT-PCR). To establish a diabetic mouse model, mice were intraperitoneally injected 150 mg/kg streptozotocin. The MDA content, ROS level and the iron level were detected by using corresponding commercial kits.

**Results:**

Maresin-1 promoted cell proliferation while reducing the apoptotic process in HG-induced ARPE-19 cells. Maresin-1 significantly reduced ferroptosis induced by HG in ARPE-19 cells, as demonstrated as a result of decreased MDA content, ROS level, Fe^2+^ level, PTGS2 expression, ACSL4 expression and increased GSH content. With respect to mechanisms, maresin-1 treatment up-regulated the mRNA expression and protein expression of HO-1, GPX4 and Nrf2 in HG-induced ARPE-19 cells. Nrf2 inhibitor reversed the inhibitory effects of maresin-1 on ferroptosis in HG-induced ARPE-19 cells. In vivo experiments, we found that Maresin-1 evidently repressed ferroptosis a mouse model of DR, as evidenced by the decreased MDA content, ROS level and iron level in retinal tissues of mice.

**Conclusion:**

Maresin-1 protects ARPE cells from HG-induced ferroptosis via activating the Nrf2/HO-1/GPX4 pathway, suggesting that maresin-1 prevents DR development.

**Supplementary Information:**

The online version contains supplementary material available at 10.1186/s12886-023-03115-9.

## Background

Diabetes retinopathy (DR), a well-known sort of diabetes complication, is the primary cause of visual loss and even blindness [1, 2]. DR is a progressive, permanent degeneration of the retinal microvasculature induced by persistent hyperglycemia [3, 4]. It is characterized by retinal edema, neuronal dysfunction and disruption of blood-retinal barrier [5, 6]. At present, DR can be alleviated via blood glucose control, but whereas blindness will inevitably occur in a considerable portion of DR patients [7, 8]. Thus, a better understanding of DR pathogenesis is necessary to seek novel therapeutic tactics.

Ferroptosis is a novel programmed mode of cell death driven by iron-dependent lipid peroxidation [9, 10]. It is caused by an imbalance between production and degradation of lipid active oxygen as well as the damage to the antioxidant capacity in cells, eventually leading to membrane rupture and cell death [11, 12]. The increased malonialdehyde (MDA) content, reactive oxygen species (ROS) level and Fe^2+^ level as well as decreased GSH content are major features of ferroptosis [13]. Recent researches have associated ferroptosis to the pathogenic process of diabetic complications, including DR [14–17]. Ferroptosis in pigmented epithelium of retina cells, in particular, is a major pattern of oxidative stress-mediated retinal pigment epithelium (RPE) cell death [18–20]. Thence, pharmacological regulation of ferroptosis has emerged as a prospective therapy option for DR [21, 22].

With the discovery of arachidonic acid-derived protectins, lipoxins and resolvins, specialized pro-resolving lipid mediators (SPMs) called maresins are discovered in lipid mediators and inflammatory exudates [23]. Maresin-1 is the first maresin to be discovered, which is a derivative of n-3 unsaturated fatty acids [24]. It is believed to have anti-inflammatory and anti-oxidative stress characteristics in acute renal failure and acute hepatic injury [25, 26]. Maresin-1, for example, alleviates the damage induced by high hyperglycemia (HG) to mice renal mesangial cells by controlling fibrosis and inflammation [27]. Maresin-1 alleviates inflammation and oxidative stress in diabetic kidney disease (DKD) [28]. Maresin-1, in particular, suppresses HG-induced ferroptosis of osteoblasts in type 2 diabetic osteoporosis (T2DOP) [29]. However, it is uncertain whether maresin-1 inhibits HG-induced cell ferroptosis in DR.

Nuclear factor erythroid 2-related factor 2 (Nrf2) is considered to be a redox-sensitive transcription factor [30]. Overproduction of intracellular ROS can promote Nrf2 translocation to the nucleus, where Nrf2 binds to downstream antioxidant response elements to induce transcriptional activation of antioxidant enzymes such as HO-1, therefore relieving cellular redox imbalance [31–33]. The pathway of Nrf2/HO-1 has been found to have a protective impact on the retina in DR [34]. Moreover, an antioxidant named phospholipid glutathione peroxidase 4 (GPX4) is used in catalytic process of peroxides for example organic hydro peroxides and hydrogen peroxides, and it prevents ferroptosis [35]. Notably, recent investigation indicated that maresin-1 may protect against ferroptosis in acute liver injury when the pathway of Nrf2/HO-1/GPX4 is activated [36]. Therefore, we tested whether maresin-1 suppressed ferroptosis in a DR cell model through activating the pathway of Nrf2/HO-1/GPX4.

In this study, we treated an adult retinal pigment epithelial cell line (ARPE-19) with HG to establish a cell model of DR, and we intraperitoneally injected streptozotocin to mice for establish a mouse model of DR. Then we explored the effect of maresin-1 on ferroptosis in these models and associated molecular pathways.

## Methods

### Cell culturing and treatments

ARPE-19 cells were bought from the American Type Culture Collection (ATCC; Manassas, VA, USA). These cells had normal karyology, and formed polarized epithelial monolayers on porous filter supports. Meantime, ARPE-19 cells had functional and structural properties resembling those of RPE cells in vivo [37]. ARPE-19 cells were cultured in Dulbecco’s modified Eagle’s medium (DMEM)/F12 (Hyclone, Logan, UT, USA) containing 1% penicillin-streptomycin (Life Technologies, Carlsbad, CA, USA) and 10% fetal bovine serum (FBS; Life Technologies). These cells were put into the incubator having 37 °C temperature, 95% humidity and 5% CO_2_. The culture medium was renewed 2-3-times every week until cells reached 80% confluence. ARPE-19 cells between passages 23 and 25 were used for the following experiments.

For exploring the role of maresin-1 (Cayman Chemical, Ann Arbor, Michigan, USA) in HG-induced ARPE-19 cells, there were total five groups:

First group: NG-group (normal glucose, 5 mmol/L glucose),

Second group: HG group (high glucose, 30 mmol/L glucose),

Third group: HG + maresin-1-L group (HG + 10 nmol/L maresin-1),

Fourth group: HG + maresin-1-M group (HG + 100 nmol/L maresin-1),

Fifth group: HG + merasin-1-H group (HG + 1000 nmol/L maresin-1).

ARPE-19 cells before exposing to HG for 24 h, they were treated with maresin-1 for thirty minutes.

In order to explore the involvement of the Nrf2/HO-1/GPX4 pathway, Nrf2 inhibitor (ML385; MCE, Shanghai, China) was used to block Nrf2 pathway activation. ARPE-19 cells were first treated with 5 μM ML385 for 48 h, followed by above mentioned maresin-1 treatment and HG treatment.

### Quantitative real time-polymerase chain reaction (qRT-PCR)

Total RNAs from ARPE-19 cells were extracted using TRIzol (TRI) reagent and extracted RNAs were quantified by using Nano Drop. After that, reverse transcription of RNA (200 ng) was performed to generate cDNA according to the guidelines of a Prime script RT reagent kit (Takara, Dalian, China). To conduct qRT-PCR, a SYBR Green PCR kit (Takara) was used, with GAPDH as the endogenous control. The following were reaction conditions: after 30 s at 95 °C, 40 cycles of 5 s at 95 °C and 35 s at 60 °C were performed. Primers were obtained from Guangzhou Boxin Biotechnology (Table 1). Relative mRNA expression of Nrf2, HO-1 and GPX4 was determined by using delta-delta CT method (2^−ΔΔCt^ method).

**Table 1:** Primer sequences for qRT-PCR in this study

Genes		Sequences (5’~3’)
Nrf2	Forward	AAACCACCCTGAAACGACAG
	Reverse	AGCGGCTTGAATGTTTGTC
HO-1	Forward	AAGACTGCGTTCCTGCTCAAC
	Reverse	AAAGCCCTACAGCAACTGTCG
GPX4	Forward	GAGGCAAGACCGAAGTAAACTAC
	Reverse	CCGAACTGGTTACACGGGAA
GAPDH	Forward	ACATCGCTCAGACACCATG
	Reverse	TGTAGTTGAGGTCAATGAAGGG

### Cell counting Kit-8 (CCK-8) test

CCK-8 assay was performed to assess proliferation of ARPE-19 cells. The 96-well plates were used for cell culture (1 × 10^4^ cells in each), which were incubated for 48 h. Then these cells were incubated with CCK-8 solution (10 μL) for 1 h. Finally, a microplate reader (Bio-Rad, CA, USA) was used to measure the optical density at 450 nm.

### Flow cytometry analysis

Apoptosis of ARPE-19 cells was evaluated by using a FITC Annexin V Apoptosis Detection Kit I (BD Biosciences, Franklin Lakes, NJ, USA). In brief, trypsin was used to harvest ARPE-19 cells, which were subsequently washed in phosphate buffered saline (PBS). These cells were re-suspended in 1 × Binding Buffer to produce a cell suspension of 1 × 10^6^ cells/mL. After transferring 100 μL cell solution to a culture tube, PI (5 μL) and FITC Annexin V (5 μL) were added. Following fifteen minutes in the dark, 1 × Binding Buffer (400 μL) was mixed and stained cells were evaluated by using Flow Cytometer (BD Biosciences).

### Examination of cell morphology

The morphological changes of ARPE-19 cells were evaluated using an inverted IX71 microscope (Olympus, Tokyo, Japan).

### Determination of Fe^2+^

The phirozine technique was used to quantify the Fe^2+^ level in ARPE-19 cells. Disintegration of ARPE-19 cells was conducted with trypsin. After centrifugation, these cells were washed twice with PBS and were lysed with RIPA lysate for 30 min. Then total proteins were extracted after centrifugation. After that, the supernatant was added to the 96-well plate (50 μL/well) and the same volume of hydrochloric acid was added. Following 30 min of reaction, the iron probe (100 μL) was added to incubate for 1 h in the dark. The absorbance at 562 nm was measured to evaluate the level of Fe^2+^.

### Detection of GSH, MDA and ROS

A colorimetric GSH test kit (Nantong, Jiangsu, China) was used to measure the GSH level. Lipid peroxidation MDA assay kit was used to assess the MDA content. The level of intracellular ROS was detected using 2’,7’-dichlorodihydrofluorescein diacetate (DCFH-DA) assay.

### Immunofluorescence staining

ARPE-19 cells were fixed via paraformaldehyde (4%), and were permeabilized via Triton X-100 (0.25%). Then these cells were blocked using bovine serum albumin (BSA) for 20 min, and primary antibodies including anti-PTGS2 (1:500, ab179800, Abcam, Cambridge, MA, USA) and anti-ACSL4 (1:200, ab155282, Abcam) were added to incubate overnight. After being washing with PBS, these cells were incubated with the anti-rabbit secondary antibody (Molecular Probes, Shanghai, China) for 1 h. The 4’,6-diamidino-2-phenylindole (DAPI; Sigma-Aldrich, Shanghai, China) was used to stain nucleic acids and a Nikon ECLIPSE E800 fluorescence microscope was used to capture the images.

### Western blotting analysis

Radio immunoprecipitation assay was used for isolating proteins of ARPE-19 cells and a BCA Protein Quantification kit evaluated the concentration of proteins. Subsequently, protein samples (40 μg) were separated using 10% sodium dodecyl sulfonate-polyacrylamide gel (Solarbio, Beijing, China), followed by moving to polyvinylidene difluoride membranes (Pall Corporation, New York, NYC, USA). After membranes were sealed via skimmed milk for 1 h, they were incubated with following primary antibodies at 4 °C overnight: anti-Nrf2 (1:500, ab62352, Abcam), anti-HO-1 (1:2000, ab52947, Abcam), anti-GPX4 (1:2000, ab125066, Abcam) and anti-β-actin (1:200, ab115777, Abcam). Then the anti-rabbit secondary antibody (1:2000, ab205718, Abcam) was added to incubate for 1 h. Finally, the bands were examined using an enhanced chemiluminescence (ECL) kit (Millipore, Billerica, MA, USA).

### Animal experiments

Total eighteen C57BL/6 mice (six-week-old, male) were bought from the Vital River company (Beijing, China). These mice were kept in a controlled environment (temperature at 20 ± 3 °C, humidity at 55 ± 5%, with a 12/12 h light/dark cycle), which had free access to water and food. The animal experiment was approved by the Animal Care and Use Ethics Committee of Beijing Viewsolid Biotechnology Co. LTD (VS232176245). All methods were carried out in accordance with relevant guidelines and regulations. All methods are reported in accordance with ARRIVE guidelines (https://arriveguidelines.org).

After acclimation for 7 days and fasting overnight, mice were intraperitoneally injected 150 mg/kg streptozotocin (STZ, Sigma, St. Louis, MO, USA) to establish a diabetic mouse model [22]. Mice with randomized blood glucose levels more than 19 mmol/L, polyuria, wasting and glycosuria were considered to have diabetes. After 3 weeks of onset of diabetes, mice were treated with maresin-1 (0.3 μg/mice) [38] via intraocular administration once a day from day 0 to day 7. During treatment, blood glucose level was monitored. After the mice were anesthetized using 2% sodium pentobarbital, the retinal tissues of mice were collected and stored at -80 °C for further experiments.

Mice were assigned into three groups (n = 6 mice/group): the control group (normal mice), the DR group (mice injected with STZ) and the DR + Maresin-1 group.

### Measurement of ferroptosis factors in vivo

The content of MDA in retinal tissues was detected via a MDA assay kit from Nanjing Jiancheng Bioengineering Institute (Nanjing, China). The level of ROS in retinal tissues was determined using an oxidation-sensitive fluorescent probe DCFH-DA with a ROS Assay kit (Jianglaibio, Shanghai, China).

For detecting the iron level, retinal tissues were homogenized in Iron Assay Buffer at 4 °C. Then the total iron in tissue homogenate was determined through an Iron Assay Kit (ab83366, Abcam). The absorbance at 593 nm was measured using a microplate reader and the iron level was normalized to the protein concentration.

### Statistical analysis

Statistical analysis was conducted through GraphPad Prism 8.0 (San Diego, CA, USA). Data from three replicates were presented as mean ± standard deviation. Difference comparisons between two groups of data were conducted via Student’s t test. Difference comparisons among multiple groups of data were conducted using one-way ANOVA, accompanied by Tukey’s test. *P* < 0.05 indicated significant differences.

## Results

### Maresin-1 promotes proliferation and impairs apoptosis in HG-induced ARPE-19 cells

**Fig. 1:**
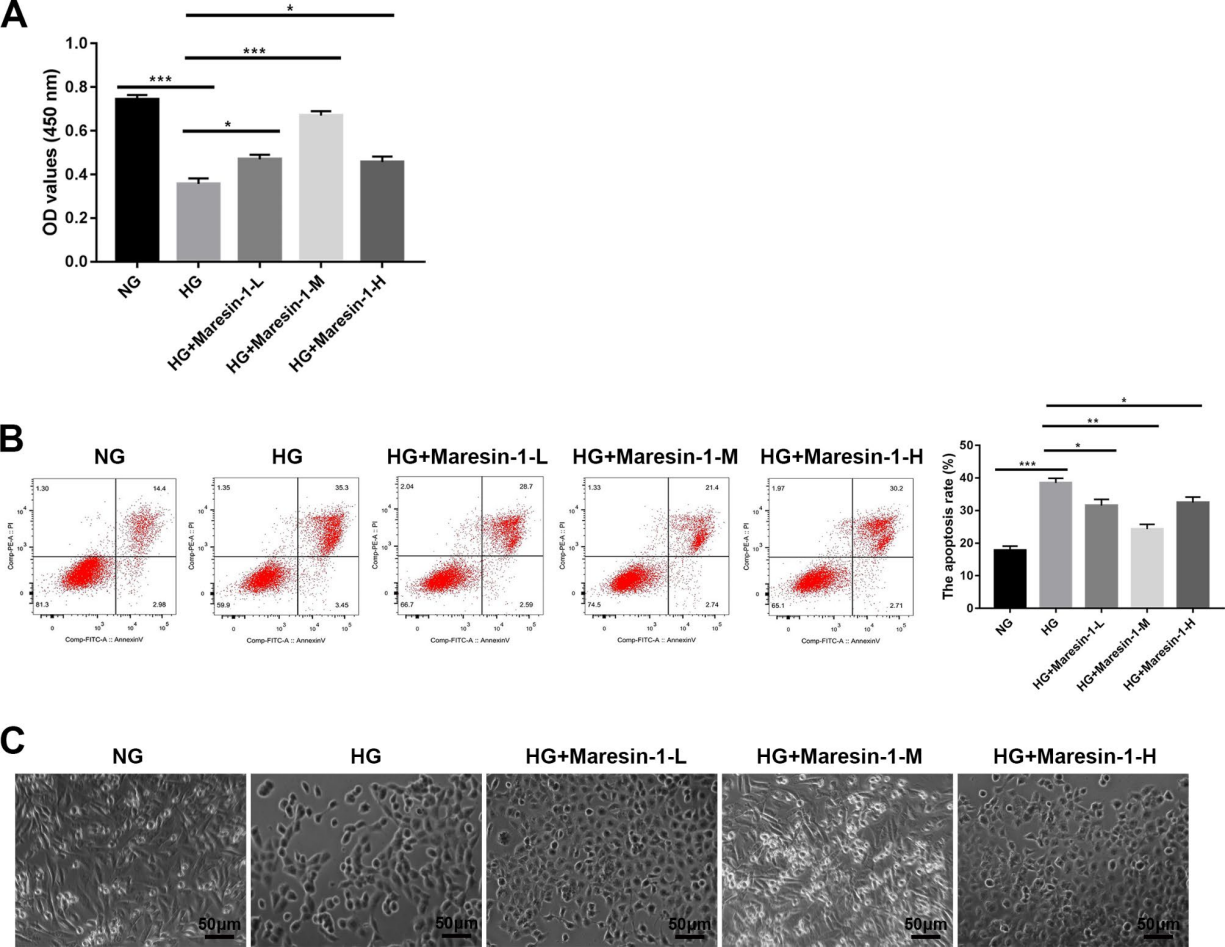
Maresin-1 promotes proliferation and impairs apoptosis in HG-induced ARPE-19 cells. (**A**) The proliferation of ARPE-19 cells was assessed by CCK-8 assay. (**B**) The apoptosis rate of ARPE-19 cells was assessed by flow cytometry. (**C**) An inverted microscope was used to observe morphological changes of ARPE-19 cells. ^*^p < 0.05, ^**^p < 0.01, ^***^p < 0.001. Each experiment was performed in triplicate in three independent experiments (n = 3)

Effects of maresin-1 on apoptosis and proliferation of ARPE-19 cells were investigated. CCK-8 assay indicated that the proliferation ability of ARPE-19 cells in the HG group was attenuated compared to the NG group (*p* < 0.001, Fig. 1A). Notably, the proliferation ability of HG-induced ARPE-19 cells was evidently enhanced by maresin-1 treatment (10, 100, and 1000 nmol/L), especially 100 nmol/L maresin-1 (*p* < 0.001, Fig. 1A). Data from flow cytometry showed that the apoptosis rate of ARPE-19 cells in the HG group was increased compared to the NG group (*p* < 0.001, Fig. 1B). Meantime, the apoptosis rate of HG-induced ARPE-19 cells was distinctly reduced by maresin-1 (10, 100, and 1000 nmol/L), especially 100 nmol/L maresin-1 (*p* < 0.01, Fig. 1B).

The influence of maresin-1 on cell morphology was investigated. ARPE-19 cells in the NG group were found to have a long spindle shape, monolayer extended adherent development, mosaic pattern, clear border, regular shape, uniform distribution, and good condition (Fig. 1C). ARPE-19 cells in the HG group, on the other hand, displayed deformation, an unclear border, disorganized organization, low cell density, and an unsatisfactory growth state (Fig. 1C). The number of round shriveled cells in HG-induced ARPE-19 cells considerably lowered after treatment of maresin-1 (10, 100, and 1000 nmol/L) (Fig. 1C).

### Maresin-1 suppresses ferroptosis in HG-induced APRE-19 cells

**Fig. 2:**
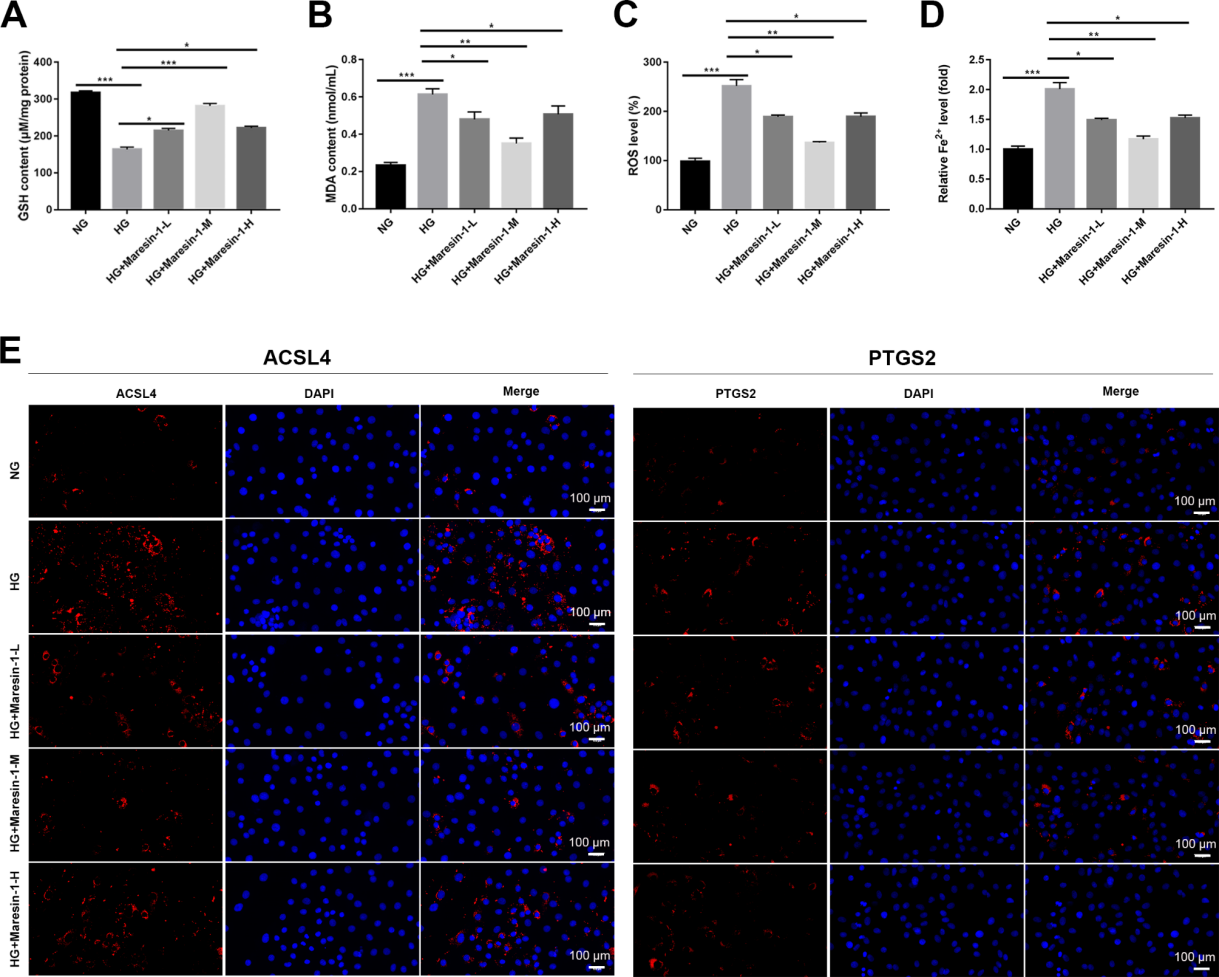
Maresin-1 represses ferroptosis in HG-induced ARPE-19 cells. (**A**) The content of GSH was determined using a colorimetric GSH assay kit. (**B**) The content of MDA was measured using a Lipid Peroxidation MDA Assay Kit. (**C**) The level of intracellular ROS was assessed using 2’,7’-dichlorodihydrofluorescein diacetate (DCFH-DA) assay. (**D**) The content of Fe^2+^ in ARPE-19 cells was determined by phirozine method. (**E**) The protein expression of ACSL4 and PTGS2 was determined by immunofluorescence staining. ^*^p < 0.05, ^**^p < 0.01, ^***^p < 0.001. Each experiment was performed in triplicate in three independent experiments (n = 3)

The influence of maresin-1 on ferroptosis was further explored by identifying ferroptosis hallmarks. As displayed in Fig. 2A and D, HG treatment accelerated ferroptosis in ARPE-19 cells, as evidenced by increased MDA level, ROS level, Fe^2+^ level, and decreased GSH level (*p* < 0.001). Simultaneously, maresin-1 administration decreased the MDA content, ROS level, and Fe^2+^ level while raising the GSH content in HG-induced ARPE-19 cells (*p* < 0.05).

Furthermore, immunofluorescence labeling was used to evaluate ferroptosis marker proteins (PTGS2 and ACSL4) [39, 40]. It was demonstrated that expression of PTGS2 and ACSL4 was higher in the HG group than the NG group (Fig. 2E). Concurrently, maresin-1 treatment dramatically reduced ACSL4 and PTGS2 expression in HG-induced ARPE-19 cells (Fig. 2E).

### Maresin-1 suppresses ferroptosis in HG-induced ARPE-19 cells via activating the Nrf2/HO-1/GPX4 pathway

**Fig. 3:**
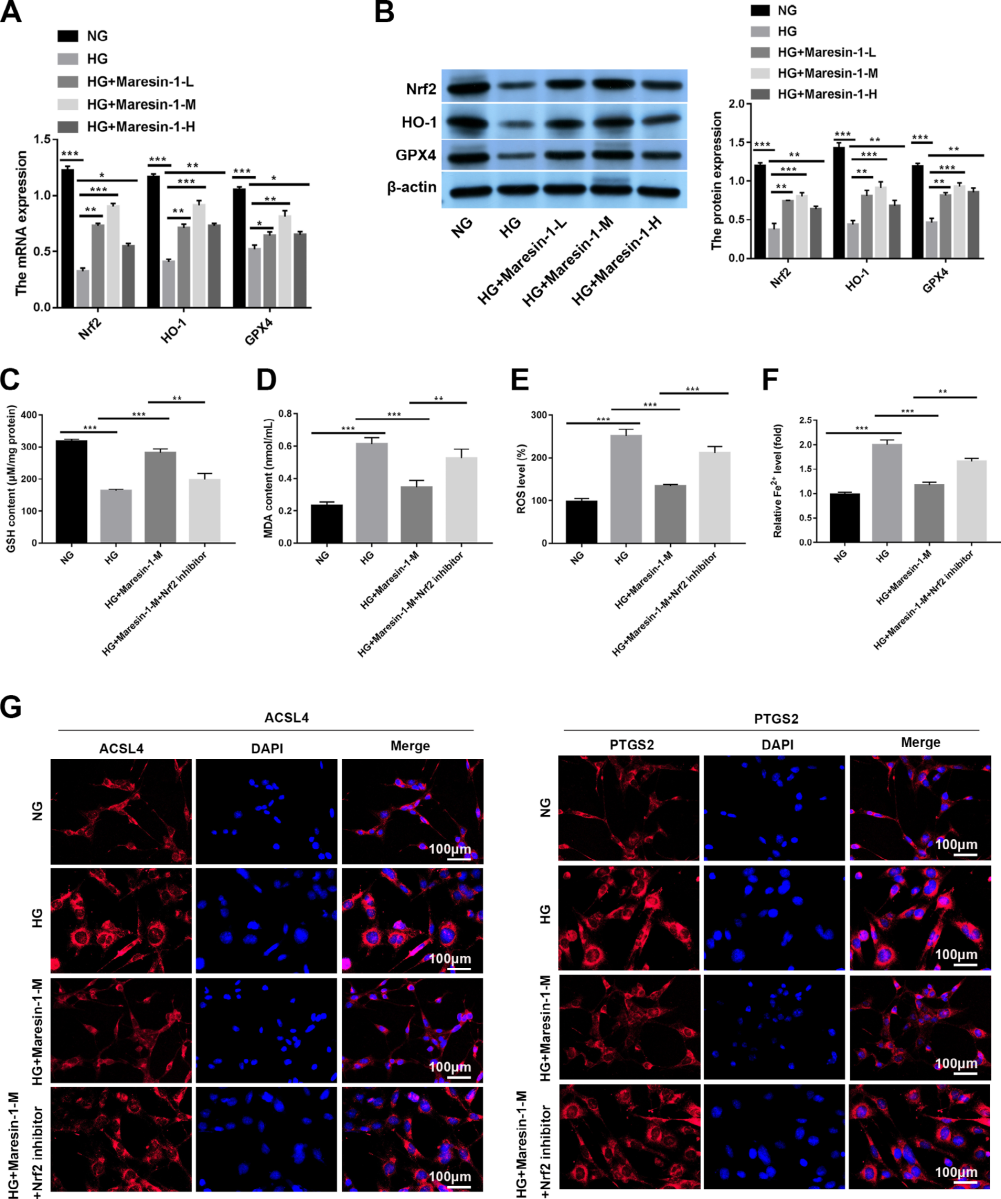
Maresin-1 activates the Nrf2/HO-1/GPX4 pathway in HG-induced ARPE-19 cells. (**A**) Relative mRNA expression of Nrf2, HO-1 and GPX4 in ARPE-19 cells was detected by quantitative real-time polymerase chain reaction (qRT-PCR). (**B**) Relative protein expression of Nrf2, HO-1 and GPX4 in ARPE-19 cells was detected by western blot. (**C**) The content of GSH in ARPE-19 cells was determined using a colorimetric GSH assay kit. (**D**) The content of MDA in ARPE-19 cells was measured using a Lipid Peroxidation MDA Assay Kit. (**E**) The level of intracellular ROS in ARPE-19 cells was assessed using 2’,7’-dichlorodihydrofluorescein diacetate (DCFH-DA) assay. (**F**) The content of Fe^2+^ in ARPE-19 cells was determined by phirozine method. (**G**) The protein expression of ACSL4 and PTGS2 in ARPE-19 cells was determined by immunofluorescence staining.^*^p < 0.05, ^**^p < 0.01, ^***^p < 0.001. Each experiment was performed in triplicate in three independent experiments (n = 3)

Finally, in HG-induced APRE-19 cells, the influence of maresin-1 on the Nrf2/HO-1/GPX4 pathway was explored. It was found that the mRNA expression and protein expression of Nrf2, HO-1 and GPX4 in the HG group were lower than those in the NG group (*p* < 0.001, Fig. 3A and B). Importantly, maresin-1 treatment increased the protein expression and mRNA of GPX4, Nrf2 and HO-1 in HG-induced ARPE-19 cells (*p* < 0.05, Fig. 3A and B). The suppressive effects of maresin-1 on MDA content, ROS level, Fe^2+^ level, PTGS2 expression and ACSL4 expression as well as the promoting effect of maresin-1 on GSH content were reversed by Nrf2 inhibitor in HG-induced ARPE-19 cells (*p* < 0.01, Fig. 3C and G). These results demonstrated that maresin-1 activated the Nrf2/HO-1/GPX4 pathway to impair ferroptosis in HG-induced ARPE-19 cells.

### Maresin-1 represses ferroptosis in a mouse model of DR

**Fig. 4:**
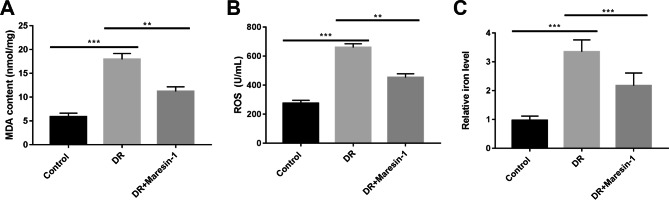
Maresin-1 represses ferroptosis in a mouse model of DR. (**A**) The content of MDA in retinal tissues was detected via a MDA assay kit from Nanjing Jiancheng Bioengineering. (**B**) The level of ROS in retinal tissues was determined using an oxidation-sensitive fluorescent probe DCFH-DA with a ROS Assay kit (Jianglaibio, Shanghai, China). (**C**) The total iron in tissue homogenate was determined through an Iron Assay Kit. ^**^p < 0.01, ^***^p < 0.001. N = 6 (mice)

To validate the effect of maresin-1 on ferroptosis in DR in vivo, a mouse model of DR was established via intraperitoneal injection of STZ. We discovered that the MDA content, ROS level and iron level were markedly boosted in retinal tissues of DR mice compared to normal mice (*p* < 0.01, Fig. 4A C). More importantly, the MDA content, ROS level and iron level in retinal tissues of DR mice were significantly decreased by maresin-1 administration (*p* < 0.01, Fig. 4A C).

## Discussion

With increasing incidence of DR, there is growing recognition of the importance of retinal protection in early diabetes [41, 42]. In consideration of sensibility of RPE cells to hyperglycemia [43, 44], we constructed a DR cell model by exposing ARPE-19 cells to HG to simulate their hyperglycemia condition. In this cell type, we discovered reduction of cell viability, increase of apoptosis and abnormal cell shape in comparison with NG-induced ARPE-19 cells. Ferroptosis is a different sort of cell death that varies from typical cell apoptosis [11]. Here, in HG-induced ARPE-19 cells, we observed increases of the Fe^2+^ level, the ROS level and MDA content as well as a decrease of the GSH level. Also, increased protein levels of PTGS2 and ACSL4 were observed. In addition, we found that the MDA content, ROS level and iron level were markedly increased in retinal tissues of STZ-induced DR mice compared to normal mice. These data definitely confirmed the occurrence of ferroptosis in this DR cell model.

Maresin-1 has previously been found to affect inflammatory regression, wound healing and tissue homeostasis [45–47]. Although the influence of maresin-1 on DR has not been investigated, the influences of maresin-1 on several diabetic disorders have been considerably documented [27, 28]. Maresin-1, for example, inhibits fibrosis and inflammation to protect mouse renal mesangial cells from HG damage [27]. Maresin-1 alleviates DKD by decreasing inflammation and oxidative stress [28]. It is noteworthy that maresin-1 regulates HG-induced ferroptosis of osteoblasts in T2DOP, which is associated with decreases in Fe^2+^, ROS, and MDA levels as well as an increase in GSH [29]. Consistently, we also discovered that maresin-1 increased the GSH level, and reduced the Fe^2+^ level, the ROS level and MDA content in HG-induced ARPE-19 cells. At same time, we found that maresin-1 reduced the PTGS2 and ACSL4 expression in HG-induced APRE-19 cells. In vivo experiments, we discovered that the MDA content, ROS level and iron level in retinal tissues of DR mice were markedly decreased by maresin-1. Taken together, our data demonstrated that maresin-1 repressed ferroptosis in HG- induced APRE-19 cells, which was in line with prior investigation. Maresin-1 may be beneficial in designing new DR treatment drugs.

Nrf2 has been found to control antioxidant protein production and to protect cells from ROS damage [48, 49]. In particular, the Nrf2/HO-1 pathway has been explored in diabetic complications [50, 51]. In diabetic kidneys, HMGB1 down-regulation protects mesangial cells from glucose-induced ferroptosis through activation of the Nrf2/HO-1pathway [14]. This pathway prevents HG-induced oxidative stress, thereby protecting the retinal pericytes [52]. In the present study, we discovered that the mRNA expression and protein expression of Nrf2, HO-1 and GPX4 were up-regulated by maresin-1 treatment in HG-induced ARPE-19 cells, suggesting activation of the Nrf2/HO-1/GPX4 pathway. At the same time, Nrf2 inhibitor reversed the inhibitory effects of maresin-1 on ferroptosis in HG-induced ARPE-19 cells. Similarly, a previous research has demonstrated that maresin-1 activates the Nrf2/HO-1/GPX4 pathway to mitigate ferroptosis-induced liver injury [36]. Our data implied that maresin-1 impaired HG-induced ferroptosis in ARPE-19 cells by activating the Nrf2/HO-1/GPX4 pathway.

There was a limitation in our study. For observing the effect of osmotic control conditions on the experiment, the mannitol control group should be established. In future studies, to assess the effect of maresin on ARPE-19 cells under high glucose exposure, the mannitol isotonic control group should be considered to negate osmotic and metabolic effects.

## Conclusions

To summarize, our data demonstrated that maresin-1 activated the Nrf2/HO-1/GPX4 pathway, consequently repressing HG-induced ferroptosis in APRE-19 cells. Also, maresin-1 inhibited ferroptosis in STZ-induced mice. Maresin-1 is suggested to be a viable therapy target for early DR.

### Electronic supplementary material

Below is the link to the electronic supplementary material.

Supplementary Material 1

## Data Availability

The data that support the findings of this study are available from the corresponding author.

## List of abbreviations

DR: Diabetic retinopathy
HG: High glucose
qRT-PCR: Quantitative real time polymerase chain reaction
