# Supplementary figures and images for: Maresin-1 inhibits high glucose induced ferroptosis in ARPE-19 cells by activating the Nrf2/HO-1/GPX4 pathway
Source: BMC Ophthalmol. 2023 Sep 6;23:368. doi: 10.1186/s12886-023-03115-9 (PMC10481498; doi:10.1186/s12886-023-03115-9)

Figure 3B


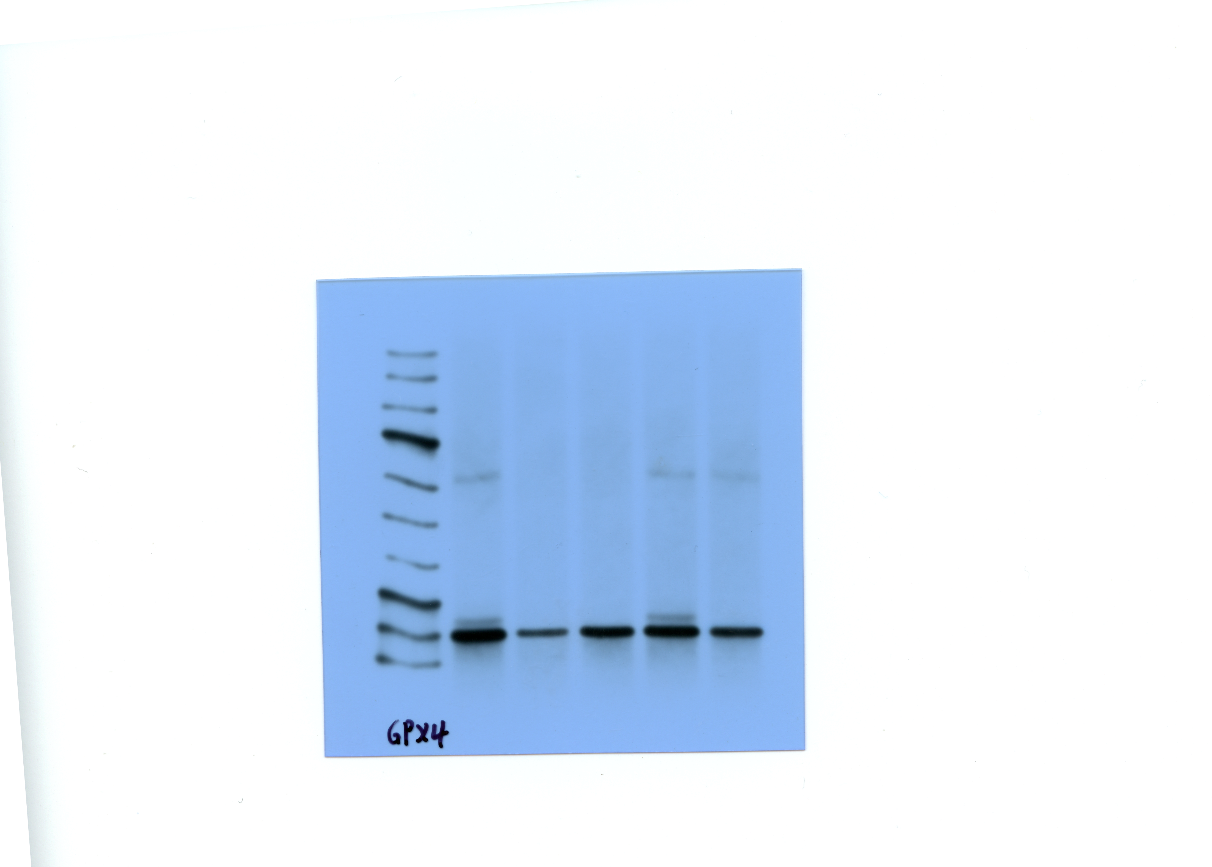

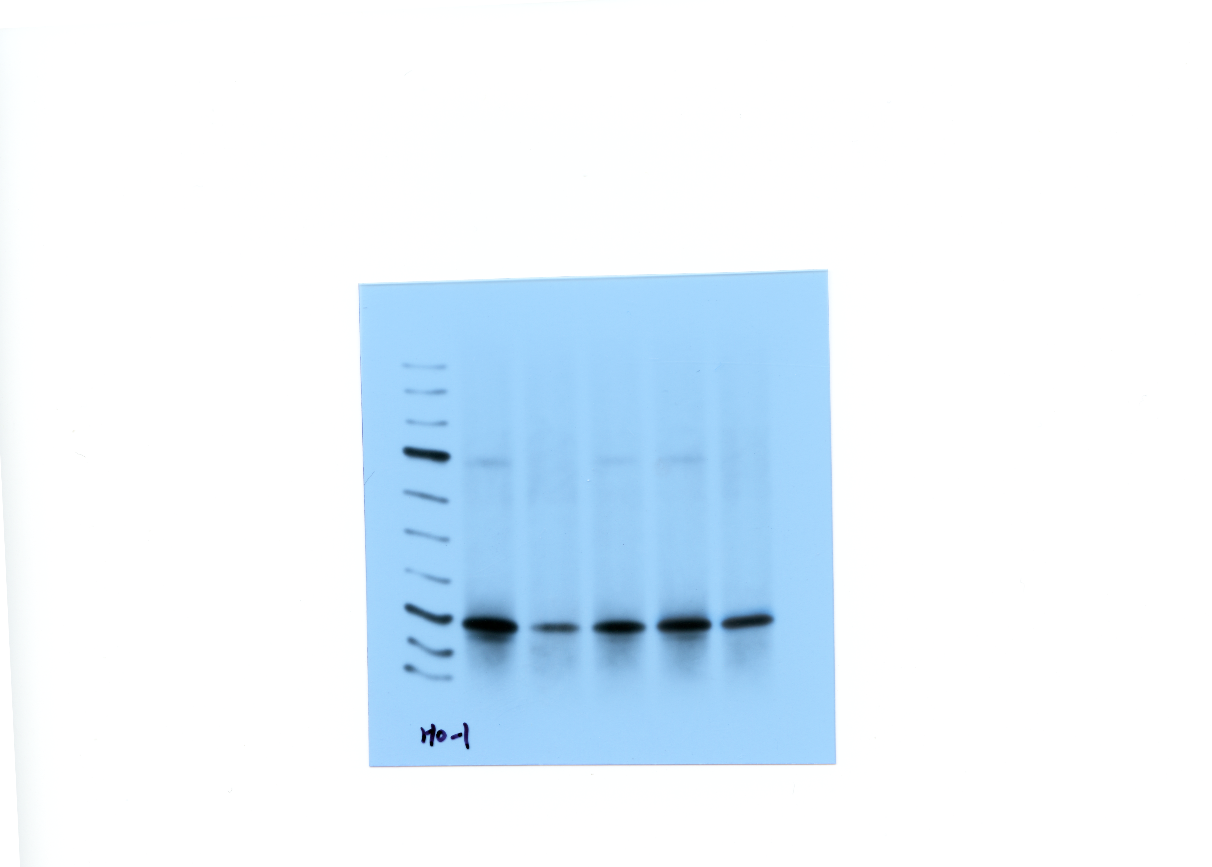

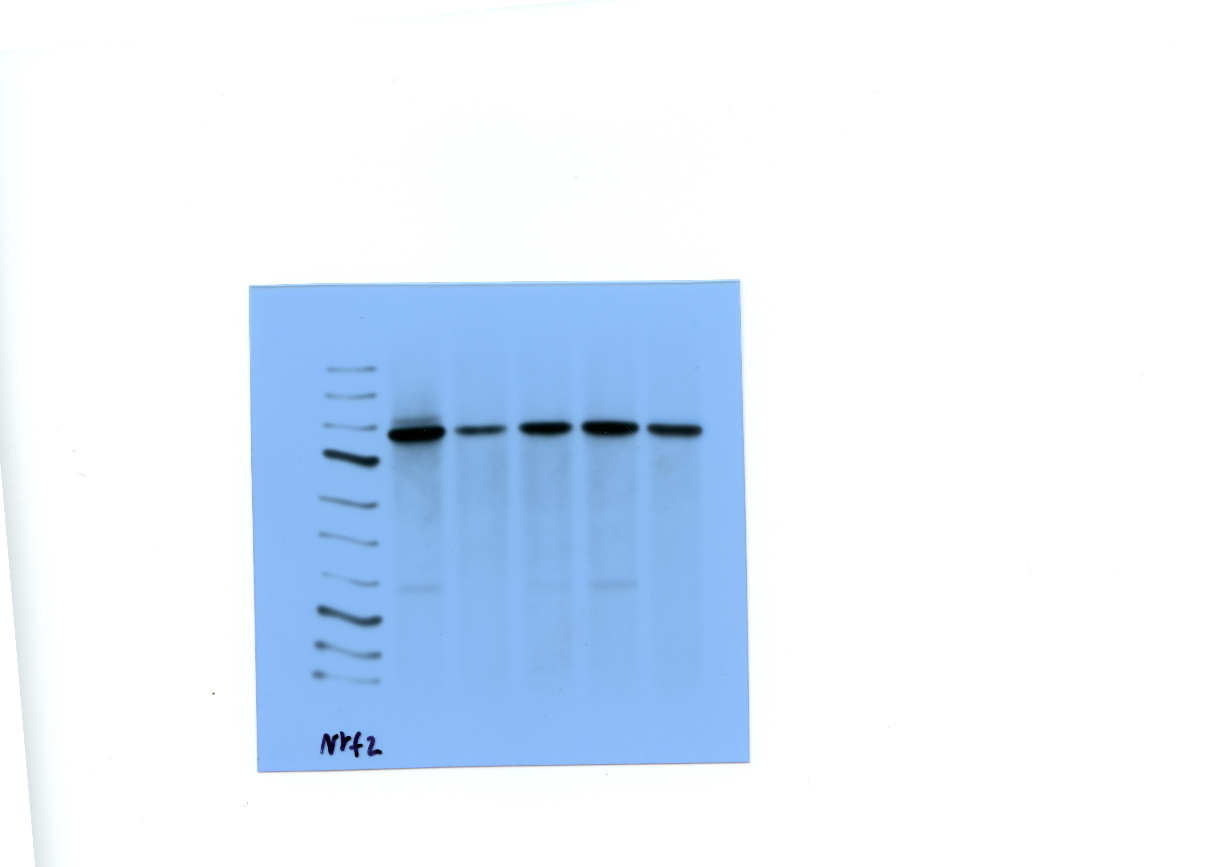

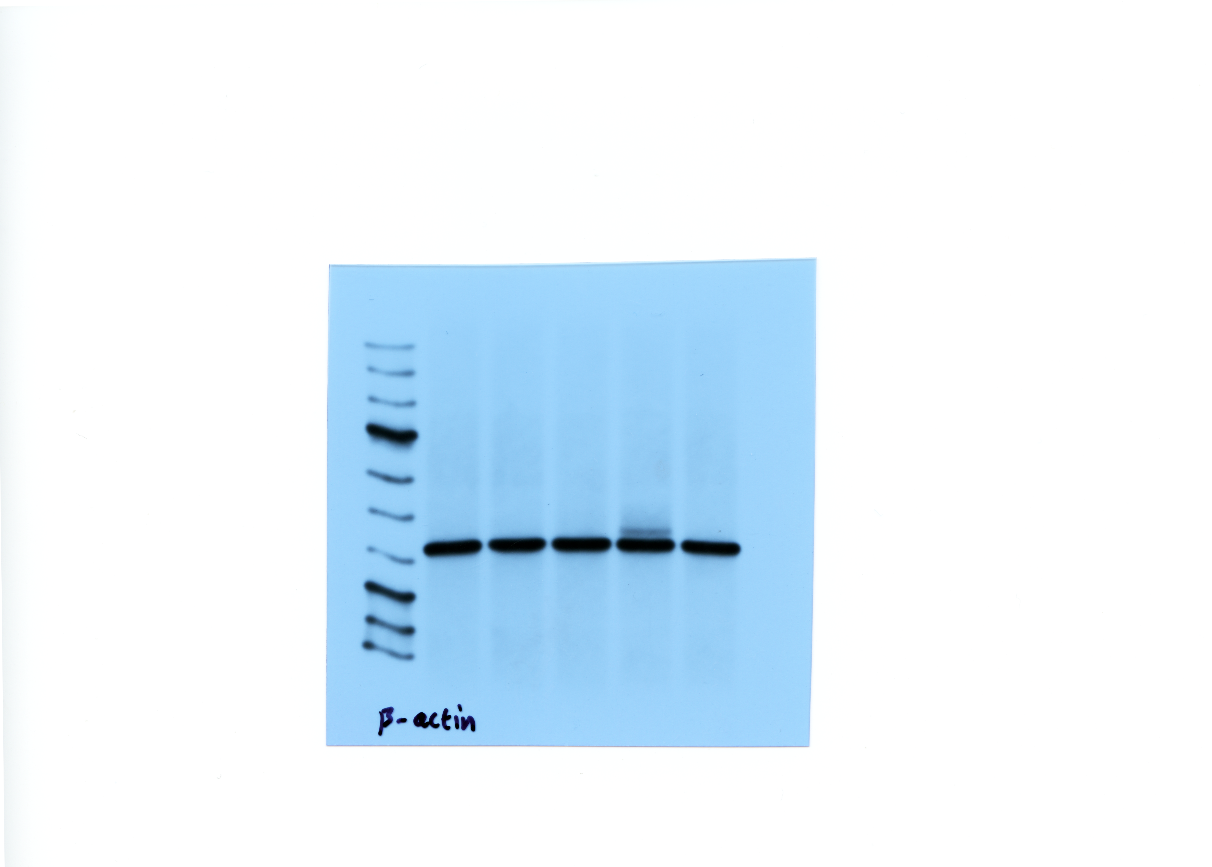

Supplement: Supplementary file 1 — Supplementary Material 1 [file 12886_2023_3115_MOESM1_ESM.docx]
